# Poorly Differentiated Non-keratinizing Sinonasal Squamous Cell Carcinoma Masquerading as Chronic Sinusitis: A Case Report

**DOI:** 10.7759/cureus.96865

**Published:** 2025-11-14

**Authors:** Yoseph M Habte, Binyam M Habte, Esimael M Abdu, Biruk T Wubneh, Shimelis A Yimer

**Affiliations:** 1 Department of Medicine, Ethio Tebib Hospital, Addis Ababa, ETH; 2 Department of Medicine, ALERT Comprehensive Specialized Hospital, Addis Ababa, ETH; 3 Department of Surgery, Teklehaimanot General Hospital, Addis Ababa, ETH; 4 Department of Medicine, University of Gondar, Gondar, ETH; 5 Department of Pathology, Ethio Tebib Hospital, Addis Ababa, ETH

**Keywords:** case report, chronic rhinosinusitis, maxillectomy, selective neck dissection, sinonasal carcinoma

## Abstract

Sinonasal squamous cell carcinoma (SNSCC) is a rare malignant tumor of the nasal cavity and paranasal sinuses, often presenting with nonspecific symptoms that can delay diagnosis. We report a case of a 50-year-old woman with a poorly differentiated non-keratinizing SNSCC of the left maxillary sinus, initially misdiagnosed as chronic sinusitis for one year. She presented with progressive nasal obstruction and facial pain. Imaging revealed an aggressive maxillary sinus lesion with bony destruction, and an initial biopsy suggested sinonasal undifferentiated carcinoma. The patient underwent left maxillectomy with selective neck dissection; final histopathology confirmed poorly differentiated non-keratinizing SNSCC with lymphovascular invasion but no nodal metastasis. Postoperative recovery was uneventful, and adjuvant radiotherapy was planned. This case underscores the diagnostic challenges of SNSCC and highlights a key clinical lesson that persistent sinus symptoms unresponsive to medical therapy should raise suspicion for malignancy. Early recognition, comprehensive evaluation, and multidisciplinary management are essential for optimal outcomes in patients with aggressive sinonasal tumors.

## Introduction

Sinonasal squamous cell carcinoma (SNSCC) is a rare malignant epithelial tumor arising from the mucosal lining of the nasal cavity and paranasal sinuses. Although it accounts for approximately 50-60% of all sinonasal malignancies, SNSCC represents only 3-5% of head and neck cancers [1-3]. The disease most commonly affects individuals in the sixth to seventh decades of life and shows a male predominance, though cases in women are also reported [4,5].

SNSCC often presents with nonspecific symptoms such as nasal obstruction, facial pain, or chronic sinusitis-like complaints, which can lead to delayed diagnosis [2,5]. Radiologic imaging with CT and MRI is crucial for assessing tumor extent, while histopathologic confirmation remains the diagnostic gold standard. Preoperative diagnostic discrepancies are not uncommon due to overlapping features with other aggressive sinonasal malignancies, such as sinonasal undifferentiated carcinoma (SNUC) [6,7].

We present a case of a 50-year-old woman with a poorly differentiated non-keratinizing SNSCC of the left maxillary sinus, initially misdiagnosed as chronic sinusitis for one year. This case report underscores the diagnostic challenges and multidisciplinary management of SNSCC and adds to the literature by highlighting prolonged misdiagnosis, biopsy-final pathology discordance, and the definitive surgical management approach.

## Case presentation

A 50-year-old woman, a housewife and retired farmer, presented to the Oral and Maxillofacial Surgery Department with facial pain, nasal congestion, and a sensation of fullness in the left nasal and upper maxillary region, persisting for one year. During this time, she was evaluated multiple times at peripheral health facilities and was repeatedly diagnosed with chronic sinusitis, receiving several courses of antibiotics and anti-inflammatory agents without improvement. Her symptoms progressively worsened, with increasing unilateral nasal obstruction and intermittent facial fullness, prompting referral for further evaluation. She had no history of tobacco or alcohol use, significant occupational exposure to industrial chemicals or wood dust, or family history of malignancy, and had no other notable comorbidities.

On physical examination, the patient appeared well nourished and in no acute distress. Vital signs were within normal limits (temperature: 36.4 °C, blood pressure: 103/71 mmHg, pulse rate: 88 bpm, and oxygen saturation: 94%). Anterior rhinoscopy revealed a soft, protruding mass occupying the left nasal cavity, causing partial obstruction. Intraoral examination demonstrated mild tenderness over the left maxillary alveolus but no mucosal ulceration or intraoral mass. No palpable cervical lymphadenopathy was identified on initial assessment.

**Figure 1:**
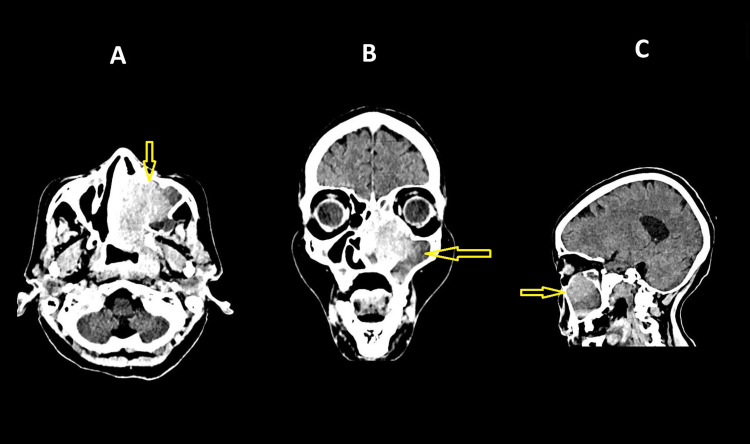
Contrast-enhanced CT of the paranasal sinuses Axial (A), coronal (B), and sagittal (C) contrast-enhanced CT images show a heterogeneous soft tissue mass (yellow arrows) occupying the left maxillary sinus, with destruction of the palatal bone and extension toward the pterygoid plate region, consistent with an aggressive sinonasal neoplasm. Based on imaging, the lesion corresponds to clinical stage cT4aN2cM0.

Contrast-enhanced CT of the paranasal sinuses revealed a heterogeneous soft tissue mass measuring 6.0 × 4.8 cm occupying the left maxillary sinus, with destruction of the palatal bone, erosion of the medial maxillary wall, nasal septum, and ethmoid septae, and involvement of the pterygoid plate region, consistent with an aggressive sinonasal neoplasm (Figure 1). The lesion extended posteriorly into the nasopharynx, causing infiltrative thickening of the nasopharyngeal mucosa. There was no radiologic evidence of orbital or intracranial extension. Neck ultrasonography demonstrated multiple suspicious cervical lymph nodes, predominantly on the left at levels II and III, the largest measuring 1.1 cm in the short axis with loss of fatty hilum, suggestive of metastatic involvement. Abdominal ultrasonography showed no visceral metastases or other abnormalities.

Routine laboratory investigations were largely within normal limits except for mild leukopenia (WBC 3.8 × 10³/µL). Serologic testing was positive for hepatitis C virus antibody and negative for hepatitis B surface antigen and HIV (Table 1).

**Table 1:** Laboratory investigations with corresponding results and reference values

Laboratory parameters	Results	Normal value
Complete blood count		
White blood cell	3.8 × 10³/µL	4.0-11.0 × 10³/µL
Hemoglobin	13.6 g/dL	12.5-15.5 g/dL
Platelet	350 × 10³/µL	150-450 × 10³/µL
Lymphocyte percentage	18.6%	15-50%
Neutrophil percentage	76%	45-80%
Metabolic panel		
Creatinine	0.8 mg/dL	0.5-1.1 mg/dL
Urea	18.2 mg/dL	17-43 mg/dL
Aspartate transaminase	43.8 U/L	2-50 U/L
Alanine transaminase	29.1 U/L	1-50 U/L
Serology		
Venereal Disease Research Laboratory test	Nonreactive	Nonreactive
Hepatitis B surface antigen	Nonreactive	Nonreactive
Hepatitis C antibody	Reactive	Nonreactive
Rapid HIV diagnostic test	Nonreactive	Nonreactive

**Figure 2:**
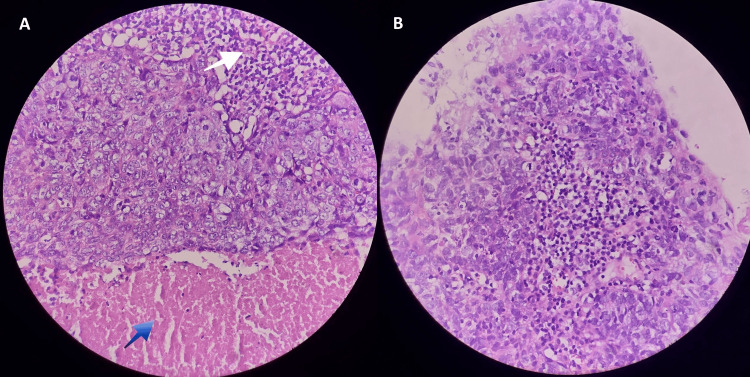
Histopathologic images of the left maxillary sinus incisional biopsy (A) Sheets of poorly differentiated epithelial cells with necrosis (blue arrow) and lymphocytic infiltrates (white arrow). (B) Poorly differentiated epithelial cells infiltrated by lymphocytes and plasma cells. Findings are consistent with undifferentiated sinonasal carcinoma.

An incisional biopsy was performed from the left maxillary sinus. Histopathologic examination showed sheets of epithelial cells with marked pleomorphism, focal squamoid differentiation, and frequent mitotic figures, consistent with undifferentiated sinonasal carcinoma (Figure 2).

Following multidisciplinary discussion, the patient underwent left maxillectomy with selective neck dissection under general anesthesia through a lip-split and lateral rhinostomy approach. The palatal mucoperiosteal flap was elevated, and the tumor was excised en bloc along with the involved soft tissue. Osteotomies were performed at the infraorbital rim, lateral nasal wall, zygomatic buttress, and palatal bone. Level I lymph node dissection with removal of the submandibular gland was completed, while level II lymph nodes were found to be adherent to major vascular structures extending toward the skull base and were therefore not resected. Hemostasis was achieved, a nasogastric drain was placed, and layered closure was performed.

**Figure 3:**
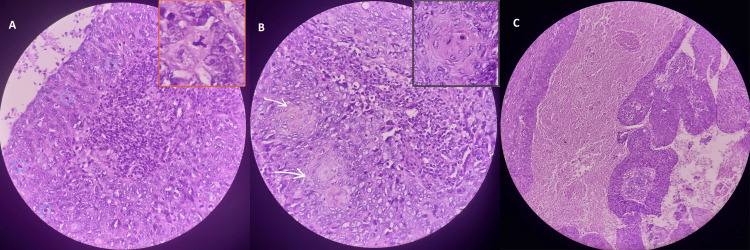
Postoperative histopathology consistent with non-keratinizing squamous cell carcinoma of the sinonasal tract (A) Frequent mitoses (blue circles) with an atypical mitotic figure highlighted in the inset. (B) Areas of squamous differentiation (arrows) with occasional keratin pearls shown in the inset. (C) Sheets and trabecular structures of pleomorphic epithelial cells in a necrotic stroma.

Gross examination of the surgical specimen revealed multiple gray-white soft tissue fragments measuring approximately 8 cm in aggregate with associated bony components. Microscopic evaluation demonstrated nests and trabeculae of malignant epithelial cells exhibiting high nuclear pleomorphism, prominent nucleoli, extensive necrosis, lymphocytic infiltration, and lymphovascular invasion. No perineural invasion was identified. These findings were consistent with a poorly differentiated (grade 3) non-keratinizing squamous cell carcinoma of the sinonasal tract (Figure 3).

The lymph node specimen contained predominantly salivary gland tissue, with one lymph node showing reactive hyperplasia and no evidence of metastasis. Because of the fragmented nature of the specimen, pathologic staging could not be determined, and clinical staging was advised based on radiologic findings.

The postoperative course was uneventful. The patient received intravenous ceftriaxone 1 g twice daily, metronidazole 500 mg three times daily, dexamethasone 6 mg twice daily, and tramadol 50 mg three times daily. She was discharged on postoperative day 4 in improved condition with oral amoxicillin-clavulanate 1 g twice daily for seven days and ibuprofen 400 mg twice daily as needed. At discharge, the surgical site was healing well, and the nasal cavity was patent with resolution of facial pain. She was referred to the oncology service for evaluation and initiation of adjuvant radiotherapy and scheduled for regular follow-up for surveillance of recurrence and functional rehabilitation.

## Discussion

SNSCC is a rare malignant epithelial neoplasm arising from the surface epithelium of the nasal cavity and paranasal sinuses, characterized by squamous differentiation [1]. It accounts for approximately 50-60% of all sinonasal malignancies but represents only about 3-5% of all head and neck cancers, with an annual incidence of roughly 0.3-1.0 cases per 100,000 population [1-3]. It has a peak incidence in the sixth to seventh decades of life [1,4]. While most epidemiologic studies describe a male predominance, our patient, a 50-year-old female, highlights that SNSCC can also occur in women, particularly in the absence of traditional risk factors such as tobacco, alcohol, or occupational chemical exposures [3,5].

SNSCC demonstrates notable biological heterogeneity. Histologically, it is classified into keratinizing and non-keratinizing subtypes, with other variants including adenosquamous, spindle cell, basaloid, papillary, and verrucous SCC, each carrying distinct prognostic implications [1,2]. In our patient, the tumor was a poorly differentiated non-keratinizing SCC with nests and trabeculae of pleomorphic epithelial cells, extensive necrosis, and lymphovascular invasion, consistent with high-grade disease and aggressive behavior. A relevant proportion of SNSCCs arise from inverted papillomas, and malignant transformation rates can reach up to 10%, emphasizing the importance of careful histologic evaluation in lesions with benign-appearing precursors [2,5].

The etiology of SNSCC remains multifactorial. While the role of tobacco is less clear than in other head and neck SCCs, occupational exposures to leather dust, chromium, arsenic, asbestos, or wood dust have been implicated [1]. Viral associations, including HPV and EBV, have been described, with HPV-positive tumors demonstrating potentially more favorable outcomes [1,3]. Our patient had no identifiable risk factors, underscoring that SNSCC can arise sporadically.

Preoperative diagnosis of sinonasal tumors is challenging due to nonspecific clinical features and overlapping histologies. Discordance between initial biopsy and final histopathology has been reported in up to 24% of sinonasal cases, highlighting the need for adequate tissue sampling and multidisciplinary evaluation [1,7]. In our case, the preoperative biopsy suggested SNUC, whereas the final postoperative specimen revealed a poorly differentiated non-keratinizing squamous cell carcinoma. This discrepancy illustrates the diagnostic complexity of sinonasal malignancies, where limited or superficial biopsy material may not fully represent the tumor’s histologic heterogeneity. The patient also had a prolonged history of misdiagnosed chronic sinusitis, which delayed definitive management, a common scenario given the subtle early symptoms of SNSCC. Imaging with CT and MRI plays a critical role in staging, particularly for assessing bony destruction, skull base invasion, orbital involvement, and potential intracranial extension [4,6]. FDG-PET/CT further assists in detecting regional metastases and may serve as a prognostic tool [6,8].

Surgical resection remains the cornerstone of SNSCC management. The choice between endoscopic and open approaches depends on tumor extent, with endoscopic techniques increasingly favored for their reduced morbidity and comparable oncologic outcomes when complete resection is achievable [2,4,6]. In our patient, a left maxillectomy with selective neck dissection via a lip-split and lateral rhinostomy approach was performed due to tumor extension into the maxillary sinus and proximity to critical structures. Despite the high-risk features of non-keratinizing histology and local aggressiveness, careful surgical planning enabled complete macroscopic resection, and no metastatic lymph nodes were identified, which aligns with the overall low propensity for nodal spread in SNSCC, particularly in nasoethmoidal tumors [1,2].

Postoperative management and adjuvant radiotherapy are recommended for high-grade, locally advanced SNSCC to improve local control and survival [6-8]. Our patient was referred for adjuvant radiotherapy and close follow-up for early detection of recurrence. This case underscores the importance of early recognition, comprehensive preoperative evaluation, and multidisciplinary management in achieving optimal outcomes in SNSCC, particularly when presentation is atypical or delayed.

## Conclusions

SNSCC is a rare and aggressive malignancy that often presents with nonspecific symptoms, leading to delayed diagnosis and management. Accurate preoperative diagnosis is challenging due to histologic heterogeneity and overlap with other sinonasal tumors. Comprehensive evaluation, including imaging and adequate biopsy, is critical for planning appropriate surgical intervention. Complete surgical resection, combined with selective neck dissection and adjuvant radiotherapy when indicated, remains the cornerstone of management. Clinically, persistent sinus symptoms unresponsive to standard medical therapy should prompt consideration of underlying malignancy. Early recognition and a multidisciplinary approach are essential to improve oncologic outcomes and minimize morbidity in patients with SNSCC, particularly in cases with atypical presentation or delayed diagnosis.
